# Transcriptome and chromatin accessibility divergence during differentiation of a bipotential progenitor cell population to erythroblasts and megakaryocytes

**DOI:** 10.1016/j.jbc.2025.110819

**Published:** 2025-10-14

**Authors:** Tejaswini Mishra, Belinda M. Giardine, Christapher S. Morrissey, Cheryl A. Keller, Elisabeth F. Heuston, Stacie M. Anderson, Vikram R. Paralkar, Maxim Pimkin, Mitchell J. Weiss, David M. Bodine, Ross C. Hardison

**Affiliations:** 1 The Pennsylvania State University, University Park, Pennsylvania, USA; 2 Department of Biochemistry and Molecular Biology, The Pennsylvania State University, University Park, Pennsylvania, USA; 3 National Human Genome Research institute, National Institutes of Health, Bethesda, Maryland, USA; 4 NHGRI Flow Cytometry Core Facility, National Human Genome Research Institute, National Institutes of Health, Bethesda, Maryland, USA; 5 Division of Hematology and Oncology, Department of Medicine, University of Pennsylvania Perelman School of Medicine, Philadelphia, Pennsylvania, USA; 6 Division of Hematology, The Children’s Hospital of Philadelphia, Philadelphia, Pennsylvania, USA

**Keywords:** transcriptomics, mRNA, cell differentiation, erythropoiesis, megakaryopoiesis, chromatin regulation

## Abstract

Changes in gene expression drive differentiation along cell lineages, and shifts in gene expression are associated with alterations in chromatin accessibility reflecting activation or repression. We used deep sequencing of polyadenylated RNA to map the transcriptomes of the megakaryocyte–erythroid progenitor (MEP) and its two daughter lineages, erythroblasts (ERYs) and megakaryocytes (MEGs), in mice to reveal insights into differentiation. Transcriptome comparisons revealed that MEPs already expressed much of the MEG program while continuing to express genes associated with parallel myeloid lineages. By contrast, ERYs underwent an extensive program of gene induction along with repression of pan-hematopoietic and MEG genes. Maps of transcription factor occupancy also indicated distinct modes of regulation for the MEG and ERY programs, with MEG genes preferentially occupied by hematopoietic transcription factors in multipotent progenitors and continued occupancy postcommitment, in contrast to erythroid genes that were primarily occupied in committed ERY. Previous work revealed a surprising discordance in the clustering of MEP with other hematopoietic cell types by RNA-Seq *versus* chromatin states. We combined the differential expression data with chromatin accessibility across blood cell types to identify trends that contribute to this discordance. Specifically, candidate *cis*-regulatory elements in some ERY-specific genes were precociously actuated in the bipotential cell populations, and some other genes were expressed in both the MEP population and MEG, but their candidate *cis*-regulatory elements have less chromatin accessibility in MEP. This discordance in cell-type clustering by different modalities of functional genomics may reflect different contributions of subpopulations in the MEP to the particular modalities measured.

Cell fate decisions are executed *via* lineage-specific gene transcription (1), but our understanding of how these processes are initiated and maintained is incomplete. Differentiation of hematopoietic stem cells into multipotential progenitors and lineage-specified cells is an excellent system for investigating the transcriptional and epigenomic landscapes during commitment and maturation (2, 3) because stem and progenitor cell populations (4, 5) can be purified using cell surface markers, and the cell lineages produced by each progenitor cell type are well characterized (6). A particularly interesting cell fate decision is the differentiation of megakaryocytes (MEGs) and erythroblasts (ERYs) from a population of megakaryocyte–erythroid progenitor (MEP) cells. This bifurcating differentiation stage is remarkable for the striking differences in the progeny cells. Committed proerythroblasts amplify by cell division (7, 8) and mature into erythrocytes with a remodeled cytoskeleton that contain large amounts of hemoglobin to facilitate oxygen and carbon dioxide transport (9). In contrast, committed megakaryoblasts undergo many rounds of DNA replication without cell division to generate polyploid MEG (10, 11) that are the source for platelet biogenesis (12). Despite the differences in maturation and function of ERY and MEG, these cell lineages can be derived from a common bipotential progenitor population, the MEP (13, 14).

The differentiation of MEG and ERY has been studied by multiple transcriptomic (15, 16) and functional genomic (17, 18, 19, 20, 21, 22, 23, 24) approaches in mice and humans. These studies revealed that differentiation of both cell types depends on shared hematopoietic transcription factors (TFs), including GATA1, GATA2, and TAL1 (25, 26), but these TFs exhibit distinctly different patterns of occupancy in the two lineages (17, 19, 20, 27), and the chromatin state maps in differentially expressed loci differ between the two lineages (21, 23, 24). In addition, ERY and MEG produce distinct TFs that facilitate their differentiation (18). For example, ETS factors, such as ERG and FLI1, are expressed preferentially in MEG and may be involved in establishing MEG-specific patterns of occupancy by GATA factors and TAL1. KLF1, an ERY-specific TF, is associated with some GATA1- and TAL1-bound DNA segments in ERYs (19, 28, 29). Furthermore, epigenomics studies using total RNA preparations and chromatin accessibility have indicated divergent modes of regulation in the two lineages (22). These studies of the population of hematopoietic stem cells termed LSK (Lin-, Sca1+, Kit+) and the common myeloid progenitor (CMP) differentiating to MEG and ERY showed that MEG retained a transcriptome similar to that of the progenitor cell types, whereas the transcriptomes of ERY reflected extensive lineage-specific induction and repression.

An open question about this bifurcation in differentiation is the relationship of MEP to other blood cell types. The placement of the population of bipotential MEP with other cell types differs depending on whether the distance measures are based on features of chromatin structure or the levels of stable RNA (Fig. 1*A*). This discordance in the groupings has been reported in multiple studies of chromatin accessibility and histone modification along with RNA across many mouse blood cell types, including both purified hematopoietic stem and progenitor cell populations and mature cells from each lineage (22, 23, 30) (Fig. S1). Specifically, MEP groups closely to erythroid cells when comparing patterns of chromatin accessibility determined by the assay for transposase-accessible chromatin followed by sequencing (ATAC-Seq) (22, 23) and in comparisons of profiles of the histone modification H3K4me1 (30). In contrast, MEP groups more closely to megakaryocytic cells and multilineage progenitors when examining stable RNA. A similar discordance in the groupings of cell types, including MEP also was observed for human blood cells (31). These differences in groupings suggest some dissociation between chromatin profiles and transcriptome profiles around the stage of the bifurcation of MEP to MEG and ERY, but a more specific explanation has not yet been established.

**Figure 1:**
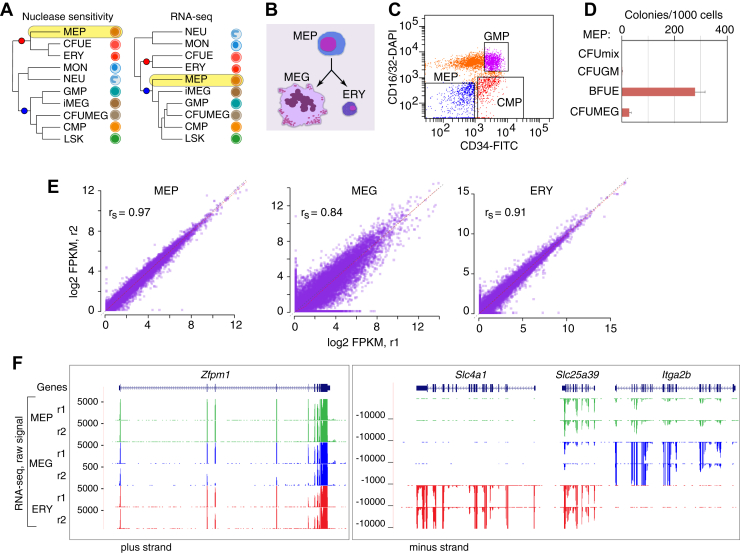
**Discordant groupings and RNA-Seq on MEP, MEG, and ERY.***A*, consensus trees of relatedness among blood cell types based on chromatin accessibility (*left*) or transcriptome (*right*) distances, summarizing published results (22, 23, 30). The placement of the megakaryocyte–erythroid progenitor cell population (MEP) is highlighted in *yellow*, and nodes that differ in the trees are marked with *red and blue circles*. *B*, relationship among cell types for which polyA+ RNA transcriptome profiling was performed. *C*, results of FACS to purify the megakaryocyte–erythroid progenitor population using CD34 and CD16/32 markers. MEPs are CD34^low^ and CD16/32(−). *D*, barplot showing the frequency of formation of colonies of distinct cell types from the purified MEP population. *E*, scatter plots of the normalized log2 FPKMs measured for RNA from the 22,977 RefSeq genes, comparing the two replicates for MEP, MEG, and ERY, along with the Spearman’s correlation coefficient (r_S_). *F*, signal profiles for the raw polyA+ RNA-Seq signal tracks from ERY, MEG, and MEP, showing only the data on the transcribed strand (synonymous with the RNA). The diagram for the *Zfpm1* gene (*left*), which is expressed in all three cell types, covers a 64 kb interval, chr8:124,802,001 to 124,866,000 on the mm9 genome assembly. The three closely linked, differentially expressed genes *Slc4a1*, *Slc25a39*, and *Itga2b* are shown using multiregion view (*right*) to focus on the intervals encompassing each, specifically chr11:102,207,001 to 102,230,000, 102,264,001 to 102,270,000, and 102,313,001 to 102,333,000, respectively. BFUE, burst-forming units erythroid; CFU, colony forming unit; CFUE, colony-forming unit erythroid; CFUMEG, colony-forming unit megakaryocyte; CMP, common myeloid progenitor; ERY, erythroblast; FACS, fluorescence-activated cell sorting; GM, granulocyte–monocyte colony; GMP, granulocyte–monocyte progenitor; iMEG, immature megakaryocyte; LSK, Lin^-^Sca^+^ Kit^+^ cells (equivalent to the murine hematopoietic stem cell population); MEG, megakaryocyte colony; mix, mixed colony; MON, monocyte; NEU, neutrophil; polyA+, polyadenylated; r1 and r2, replicates 1 and 2.

Transcriptome profiling in single cells across hematopoietic differentiation in mouse and human revealed that the multipotent and bipotent progenitor cells are not homogeneous cell populations, but rather, they represent major transitional stages along a largely continuous trajectory of differentiation (32, 33, 34). The human MEP population has been shown to consist of at least three subpopulations, all of which have a component of bipotential cells. A majority of one subpopulation are bipotential cells, and the other two subpopulations are biased toward either erythroid or megakaryocytic differentiation (34, 35). Identification of bipotential MEP is challenging for many reasons, including the much higher proliferative potential of ERY than MEG and the fact that MEG and ERY require different culture conditions, which confounds assays for both cell types simultaneously (36). The single-cell data suggest a common ancestor for progenitor cells committed to the erythroid and megakaryocytic lineages, but since there is no culture system that can produce mixed erythroid and megakaryocytic colonies, the common ancestor should be considered a model rather than a demonstrated cell type. Indeed, controversy persists over which purification schemes are best for MEP in mouse and human (36), but some methods for isolating MEP have been widely used (37).

We reasoned that some of the controversy about the MEP bifurcation in blood cell differentiation could arise from lower coverage RNA-Seq in the rare progenitor cell populations and especially in the sparse coverage for single-cell assays. Such low coverage could lead to missing some informative transcripts. To overcome the limitations of sparse coverage, we turned to deeply sequenced, polyadenylated (polyA+) transcriptomes from mouse MEP, ERY, and MEG that had been previously analyzed for long noncoding transcripts in these lineages (38). The experiments generating these datasets analyzed transcriptomes of MEP, MEG, and ERY using directional RNA-Seq to a very deep coverage, over 100 million reads per replicate (38). Thus, these data should reveal even rare transcripts. In this article, we identify the transcriptional signatures characteristic of the bipotential progenitor population and examine the nature and extent of similarities and differences between the MEP compared with its MEG and ERY progeny. Our findings confirm a high similarity in transcriptomes between MEP and MEG, whereas, in contrast, execution of the ERY program involves active induction and repression of specific genes. Further analysis of ATAC-Seq data suggests a resolution for the inconsistent grouping of MEP based on transcriptomes *versus* chromatin. A subset of the genes that are strongly upregulated in ERY show evidence of open chromatin at their candidate *cis*-regulatory elements (cCREs) in MEP, whereas a subset of genes associated with multipotent progenitors that continue to be expressed in MEG have lower ATAC-Seq signals in MEP. These patterns contribute to the clustering of MEP and MEG with other progenitors by RNA-Seq but the clustering of MEP with ERY by chromatin accessibility.

## Results

### RNA-Seq in MEP, MEG, and ERY

The levels of polyA+ RNAs in ERY, MEG, and MEP (Fig. 1*B*) were measured using directional RNA-Seq. ERY and MEG were isolated from mouse fetal liver, and MEPs were isolated from adult mouse bone marrow by fluorescence-activated cell sorting (Fig. 1*C*). MEPs formed megakaryocytic and erythroid colonies almost exclusively (Fig. 1*D*). After performing strand-specific RNA-Seq (39) on two biological replicates for each sample, the reads were processed through Cufflinks and Cuffdiff (40, 41, 42, 43) to obtain measures of expression levels (Fig. S2) for 22,977 RefSeq genes, including genes coding for proteins as well as known noncoding RNAs in the three cell types. The replicates were highly concordant in expression levels (nonzero log2 Fragments Per Kilobase of exon model per Million mapped fragments [FPKMs]), with Spearman’s correlation coefficients of 0.97, 0.84, and 0.91 for MEP, MEG, and ERY, respectively (Fig. 1*E*). The quality and reproducibility of the RNA-Seq data are illustrated for the MEG- and ERY-expressed *Zfpm1* gene (encoding FOG1) and for lineage-specific genes, *Slc4a1* (encoding the ERY anion transporter band 3), *Slc25a39* (encoding a mitochondrial glutathione transporter), and *Itga2b* (encoding the MEG integrin alpha 2b) (Fig. 1*F*).

### Distributions of gene expression during erythromegakaryopoiesis

**Figure 2:**
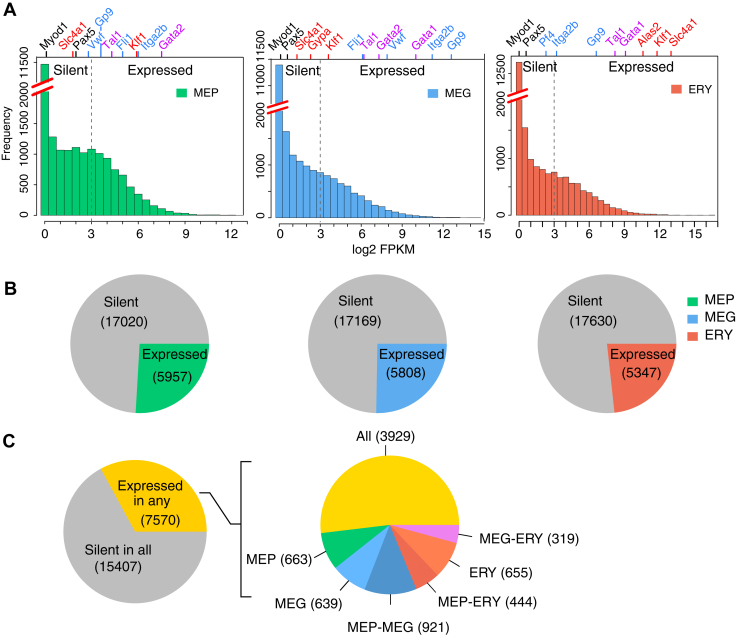
**Distributions of gene expression during erythromegakaryopoiesis.***A*, histograms showing the distribution of gene expression levels (log2 FPKM for pooled replicates plotted in bins along the *X*-axis) in the three cell types. Histograms are gapped along the *Y*-axis to better display the frequencies of moderately and highly expressed genes. Marker genes are indicated along the top *X*-axis, lined up at their level of expression on the bottom *X*-axis. Colors of gene names indicate lineage affiliation; ERY genes are in *red*, MEG genes in *blue*, genes common to both are in *violet*, and nonmyeloid or nonhematopoietic genes are in *black*. The *dotted line* at log2 FPKM = 3 indicates an empirically determined threshold for reliable detection of expression. *B*, Pie charts showing the numbers of genes transcribed in each lineage. *Gray* indicates the number of silent genes and a *nongray color* (MEP = *green*, MEG = *blue*, and ERY = *red*) indicates number of expressed genes. *C*, Pie charts showing the extent of shared and lineage-specific transcription. The pie chart on the *left* shows the number of genes expressed in any lineage. The pie chart on the *right* shows the distribution of the 7570 expressed genes across one, two, or all three lineages. ERY, erythroblast; FPKM, Fragments Per Kilobase of exon model per Million mapped fragment; MEG, megakaryocyte; MEP, megakaryocyte–erythroid progenitor.

Within each cell type, most genes were expressed at low or undetectable levels, whereas a minority of genes were expressed at moderate to high levels (Fig. 2*A*). The expression levels for genes from other cell lineages were used to define the threshold for assigning a gene as expressed. Specifically, the RNA-Seq fragments mapping to the lymphoid gene *Pax5* and the muscle determination gene *Myod1* gave a signal below a log2 FPKM of 3 in these deeply sequenced samples (Fig. 2*A*), and thus, we considered genes with expression levels at or below this threshold to be silent. Using this conservative, stringent threshold allowed us to focus on the robustly expressed genes in each cell type.

Applying this threshold, we estimate that 23% to 25% of the genes were expressed in each cell type (Fig. 2*B*). We identified 7570 genes that were expressed in at least one of the three cell types (Fig. 2*C*, *left*). About a quarter of those (1957 genes) were expressed in only one of the three cell types, divided almost equally among the three (Fig. 2*C*, *right*). About half (3929 genes) were expressed in all three cell types, indicating a large sharing of the transcription program among the progenitor and both daughter lineages. Considering genes expressed in two cell types, it is notable that MEP shared a larger number of expressed genes with MEG (921) than with ERY (444). Thus, the MEP transcription program includes genes also expressed in the daughter lineages, and substantially more (χ^2^ *p* value < 2.2e-16) of them are expressed in MEG than in ERY.

### MEPs are more similar transcriptionally to MEGs than to ERYs

**Figure 3:**
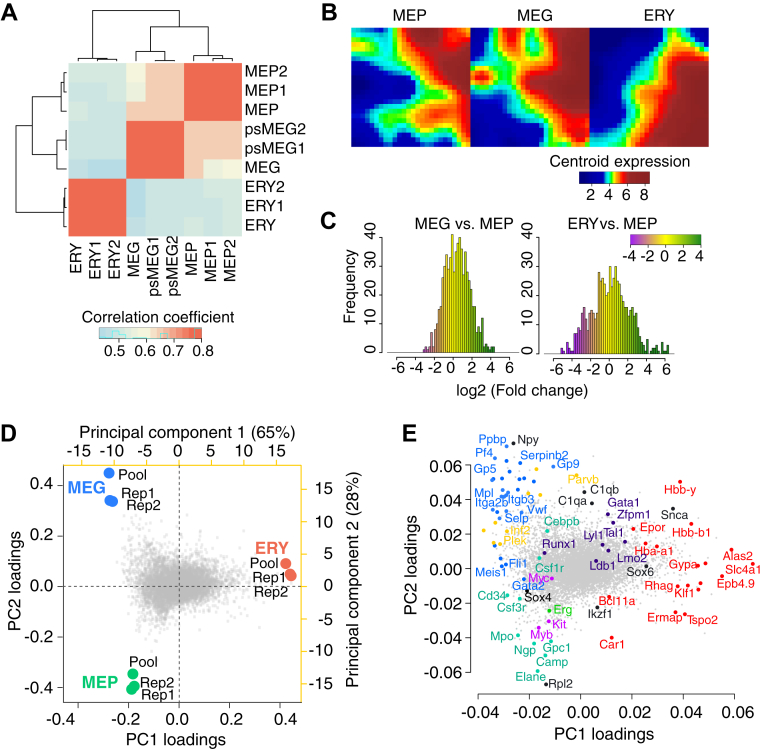
**Global relationships among MEP, MEG, and ERY transcriptomes.***A*, hierarchical clustering of replicate expression levels from MEP, MEG, and ERY based on Pearson’s correlation coefficient as a distance metric. Dendrograms indicate the relationship between the cell types. Both replicate (numerical suffixes 1 and 2) and pooled data (no suffix) are shown, with pseudoreplicates being used for MEG (psMEG). *B*, self-organizing maps (SOMs) comparing expression trends across cell types generated using the GEDI tool. Groups of genes with similar expression patterns across cell types are spatially clustered together. Each tile representing a minicluster of genes is colored by its centroid expression level. *C*, histograms depicting distribution of fold changes of tiles in differential SOMs for MEG *versus* MEP and ERY *versus* MEP. Colors indicate level of signed fold change. *D*, cell lineages were clustered using principal component analysis based on replicate (Rep1 and Rep2) and pooled expression levels in each of the 7570 expressed genes, and the results were graphed as a biplot. On the *bottom X*-axis and *left Y*-axis (*black*), the biplot reflects the individual contribution of each gene (loadings, in *gray dots*) toward principal components (PCs) 1 and 2. The *top X*-axis and *right Y*-axis (in *yellow*) reflect the PC scores of each sample (*red*, *green*, and *blue circles*). PC1 separates MEP–MEG from ERY, and PC2 separates MEP from MEG. *E*, informative individual genes contributing to these PC scores are labeled at the dot corresponding to the loadings on PC1 and PC2. *Blue dots* indicate MEG genes, *yellow dots* are for cytoskeletal proteins, *teal dots* indicate genes of non-MEG–ERY myeloid lineages, *purple* indicates genes for both MEG and ERY lineages and *red* indicates ERY genes. ERY, erythroblast; MEG, megakaryocyte; MEP, megakaryocyte–erythroid progenitor.

We compared the global transcriptomes of MEP, MEG, and ERY using three independent approaches. First, we grouped cell types by unsupervised hierarchical clustering, using correlation coefficients (Pearson’s *r*) between transcriptome profiles of all 7570 expressed genes as a distance metric. To reduce the impact of the lower correlation between MEG replicates on the overall hierarchy, we used pseudoreplicates from pooled reads of the replicates (see the Experimental procedures section). We found that the MEP grouped together with MEG to the exclusion of ERY (Fig. 3*A*), showing that the transcriptome profile of MEPs was globally more similar to that of MEG than ERY.

We then used a machine-learning method to compare genome-wide expression profiles. Self-organizing maps (SOMs) generated with the GEDI tool (44) illustrated broad expression trends across lineages. The SOMs were built from miniclusters of coexpressed genes whose spatial locations were preserved across maps of each cell type (45). Thus, the SOMs allow direct comparisons between expression levels of specific groups of genes and reveal the overall degree of differences between maps. Each tile in an SOM corresponds to the same group of genes across maps, and the color of the tile reflects the centroid expression level of its group of genes (Fig. 3*B*). Tiles with similar expression levels across cell types were spatially clustered together. The patterns in the SOMs showed that the MEP and MEG were more similar to each other than either was to ERY (Fig. 3*B*). Differential maps depicting the log of the fold change between lineages showed this trend quantitatively (Fig. 3*C*), with more tiles in ERY than MEG with large changes in expression when compared with MEP.

Finally, we examined the global relationships among the cell types using principal component analysis (PCA). The PCA projected our transcriptome data onto a new coordinate system of axes called principal components (PCs), which for each cell type was a linear combination of the expression level of each individual gene. Since the first few PCs can explain a large amount of variability in the data, this method reveals the most informative relationships among cell types while reducing dimensionality and retaining information on the contribution of each individual gene. The PCA results (Fig. 3*D*) show groupings of the lineages along the first and second PCs as well as the contribution of each of the 7570 genes (loadings) to these groupings. Replicate and pooled samples belonging to each cell type clustered faithfully for each lineage. PC1, the axis that explained the largest amount of variance in the data (65%), separated ERY from MEG and MEP. The fact that the major component to the variance in expression for all genes separated ERY from the other two cell types shows again that MEPs are closer in expression pattern to MEG. The second principal component (PC2) explained an additional 28% of the variance and separated MEG from MEP.

As expected, genes with high positive loadings along PC1, which separates ERY from MEP–MEG, were well-known erythroid markers (*red dots*, Fig. 3*E*). Genes with negative loadings on PC1 contributed to the MEP and MEG groupings. Those with positive loadings on PC2 contributed more to the MEG lineage than to MEP, and they included well-known megakaryocytic genes. Strikingly, several genes contributing to the MEP lineage (*i.e.*, with negative values along PC1 and PC2) were also highly expressed on other myeloid cells, such as granulocytes and macrophages. Examples include genes encoding myeloperoxidase (*Mpo*), neutrophil elastase (*Elane*), and neutrophil granule protein (*Ngp*). We conclude from the results of three independent computational approaches analyzing the polyA+ RNA that the transcriptome of MEPs was more closely related to MEG than ERY.

### Erythroid transcription program undergoes a greater degree of upregulation

Since the MEP transcriptome preferentially expresses MEG genes relative to ERY genes, we hypothesized that erythroid genes would need to be activated in ERY relative to their expression in MEP. We tested this hypothesis by examining the changes in gene expression after MEPs differentiate into MEG and ERY. To ensure that our results are robust, we used two different methods to characterize differentially expressed genes, Cuffdiff (41, 42) and k-means clustering, evaluating the hypothesis in both sets of results as well as a consensus set.

**Figure 4:**
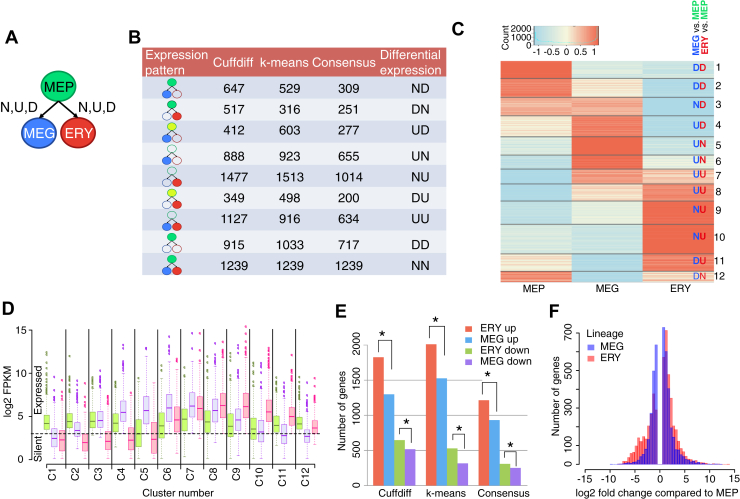
**Erythroid program is characterized by a greater degree of induction.***A*, schematic showing the pairwise differential expression tests performed for MEG (*blue oval*) and ERY (*red oval*) using MEP (*green oval*) as a reference. Possible outcomes are induction (“U”), repression (“D”), and no change (“N”). *B*, the table showing the number of genes declared differentially expressed using each method (Cuffdiff and k-means) and their consensus. The *cartoons* indicate expression patterns: *red-, blue-, or green-filled ovals* indicate expression in ERY, MEG, and MEP, respectively. The absence of a color (*empty ovals*) indicates no expression in a lineage. *C*, heatmap depicting genes clustered based on expression level in each of the three cell types. *Red* = relatively higher expression and *blue* = low expression, as represented by row-standardized log2 FPKMs. *D*, distributions of unstandardized log2 FPKMs are shown for genes in each cluster. The *dotted line* indicates the threshold for assessing that a gene was expressed. *E*, barplots summarizing the number of induced and repressed genes in ERY and MEG, as compared with MEP. Gene categories are “ERY up” (DU + NU), MEG up (UD + UN), ERY down (ND), and MEG down (DN). *Stars* indicate that differences between categories were significant using the Chi-squared test, with Bonferroni-corrected *p* values ranging from 0.04 to 6 × 10^−16^. *F*, histograms showing the distribution of differentially expressed genes by their fold change in ERY or MEG as compared with MEP. ERY, erythroblast; FPKM, Fragments Per Kilobase of exon model per Million mapped fragment; MEG, megakaryocyte; MEP, megakaryocyte–erythroid progenitor.

First, pairwise differential expression tests using Cuffdiff compared each differentiated cell type to the MEP as a reference. Genes were declared significantly changing if they met both the criteria: (i) their differential expression passed a false discovery rate (FDR) threshold of 0.05 and (ii) they were expressed in at least one of the two lineages being compared. Based on the direction of change, genes were categorized as upregulated (U), downregulated (D), or not changing significantly (N) in the differentiated cells as compared with the progenitor (Fig. 4, *A* and *B*). The upregulated category includes both genes activated from a “silent” state in MEP to “expressed” in the daughter cells and genes expressed at a low level in MEP but induced to a higher level in the daughter cells. A composite notation conveyed differential expression in both lineages; for example, “UN” denotes a gene upregulated in MEG and not changing significantly in ERY, as compared with MEP. This process assigned 6331 differentially expressed genes to nine such expression-change categories.

We then used k-means clustering to classify the differentially expressed genes into categories. This approach differs from the pairwise comparisons in Cuffdiff because it considers expression levels across all three cell types simultaneously. Varying k, the number of clusters, from 1 to 25 showed that at k = 12, the within-cluster sum of squares was minimized (Fig. S3), and hence, the genes were tightly clustered. The 12 clusters grouped informative subsets of genes by expression pattern across the three cell types, revealing both lineage-specific and lineage-shared genes (Fig. 4, *C* and *D*).

Despite showing similar trends for the categories of differentially expressed genes, Cuffdiff and k-means clustering placed different numbers of genes in each category (Fig. 4*B*). Therefore, we generated a third, consensus set of 4057 differentially expressed genes, limiting the genes in each category to the ones that were assigned to the same category by both methods (Fig. 4*B*). The expressed genes, their log2 FPKM in each cell type, and their differential expression cluster assignments are provided in Table S1.

All three sets of differentially expressed genes strongly support the hypothesis that induction is a dominant mode of regulation in erythroid differentiation. Considering the genes with expression changes in the MEG and ERY lineages, the number induced when MEPs differentiate into ERY is consistently higher than the number induced when MEPs follow the alternate fate to MEG (Fig. 4*E*) in all three sets. Also, the number of genes repressed as MEPs differentiate to ERY was greater (Fig. 4*E*, Bonferroni-adjusted χ^2^ *p* values < 0.05 for all sets) than the number repressed in MEP. Furthermore, a larger number of genes showed higher and lower levels of expression change during differentiation to ERY than to MEG (Fig. 4*F*, Kolmogorov–Smirnov test *p* value < 2.2e-16). These results show that a larger number of genes are actively regulated, mainly by induction, in erythropoiesis than in megakaryopoiesis after the MEP stage.

### Enrichment of function-related terms in lineage-specific and shared genes

**Figure 5:**
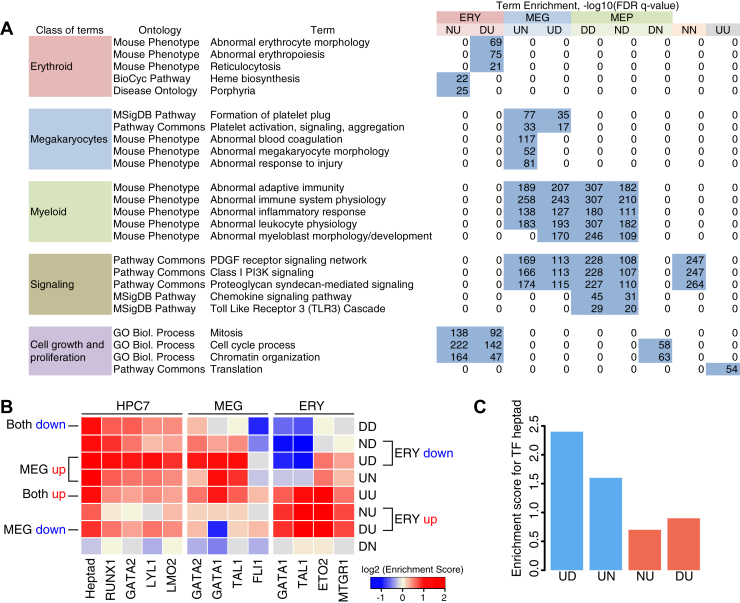
**Functional term enrichments and transcription factor (TF) occupancy for expression categories.***A*, function-related terms that were significantly enriched (binomial and hypergeometric FDR <0.05) in the gene sets for each differential expression category were determined across multiple ontologies using GREAT (46). Examination of the 50 most significant terms for each expression category revealed frequent terms in five classes of terms, which are shown along with illustrative specific terms and the negative logarithm of the FDR *q* values in each expression category, with nonzero values highlighted. *B*, heatmap showing the enrichment of TF occupancy (*columns*) in genes in each expression category (*row*). TF peaks were assigned to a gene if they were found in the gene neighborhood, defined as the interval from 10 kb upstream of the transcription start site (TSS) to 10 kb past the polyA addition site (20). Each gene can thus be assigned multiple peaks, and peaks could also be assigned to multiple genes. Fold enrichment (or depletion) was computed as the percentage of genes in an expression category occupied by each TF divided by the percentage occupied in a background set and expressed as the log_2_-fold enrichment (represented by the color of each tile). *C*, the extent of binding of the TF heptad in HPC7 cells to genes in selected differential expression categories, represented as the enrichment scores computed for *B*, is shown as bar plots to facilitate quantitative comparison. TF heptad enrichment scores for genes that were upregulated during MEP to MEG differentiation are presented as *blue bars*, and the scores for genes that were upregulated during MEP to ERY differentiation are shown as *red bars*. ERY, erythroblast; FDR, false discovery rate; MEG, megakaryocyte; MEP, megakaryocyte–erythroid progenitor.

We used the GREAT computational tool (46) to investigate more completely which functions were enriched in the genes in the nine expression categories (Fig. 5*A*). As expected, the annotations of genes upregulated specifically in ERY, that is, expression categories NU and DU, were enriched in terms related to erythroid functions such as erythrocyte morphology and heme biosynthesis. The ERY-specific genes were also enriched in terms associated with cell growth and proliferation, consistent with the rapid cell division that occurs in early erythroid differentiation. Likewise, the annotations of genes upregulated specifically in MEG, that is, expression categories UN and UD, were enriched in terms related to MEG functions such as platelet activation and blood coagulation (Fig. 5*A*).

We were particularly interested in the functions of the genes expressed in MEP but that were downregulated in both MEG and ERY, that is, category DD. The annotations for these genes were highly enriched in terms associated with other myeloid cells, for example, adaptive immunity and inflammatory response, as well as several signaling pathways, such as platelet-derived growth factor receptor signaling and PI3K signaling (Fig. 5*A*). Notably, these term enrichments were also observed for genes expressed in MEG, both those that were upregulated during differentiation to MEG (UN and UD categories) as well as those that were expressed in MEP and retained in MEG (ND category). We conclude that the expression profile of the MEP population contains genes characteristic of myeloid cells, including those encoding proteins of several signaling pathways. Expression of some of these genes is retained in MEG, but they are not expressed in ERY.

### TF occupancy in differentially expressed genes

Differential expression analysis showed that a substantial number of genes were coexpressed in MEP and MEG, whereas another large set was induced specifically in ERY. We hypothesized that differences in TF occupancy could explain, at least in part, these distinctive expression patterns, and we investigated the predicted association by calculating enrichment of the genes for chromatin occupancy by key TFs in relevant cell types. Specifically, we used chromatin immunoprecipitation sequencing (ChIP-Seq) peak calls for TF occupancy in a cell line model for multipotent hematopoietic progenitor cells (*e.g.*, HPC7) (47), in MEG (19), and in erythroid cells (19, 48) (peak statistics and sources are provided in Fig. S4). The data from HPC7 cells were used as a proxy for MEPs since the latter could not be obtained in sufficient numbers to perform standard ChIP-Seq assays. Validation experiments in transgenic mice have shown that data from this cell line provide informative insights into regulation (47). While in the context of our study, we used the data from HPC7 cells as a proxy for TF occupancy in MEP; the ChIP-Seq data indicate likely binding in early progenitors of myeloid cells, such as CMP, but also including MEP.

Enrichment or depletion of occupancy by each TF for genes in each expression category was computed based on the numbers of TF occupancy peaks observed in each gene neighborhood (the entire gene with an extension of 10 kb on each end) compared with the numbers of peaks in the set of 1239 genes with no change in expression. This analysis considers numbers of occupied sites in the genes, but it does not investigate or compare which regulatory elements in the genes are bound. Some elements could be bound by different sets of TFs in different cell types.

Genes in almost all the differential expression categories were enriched for occupancy by TFs in the progenitor cell line model HPC7 (Fig. 5*B*). The exceptions were genes in the DN category, which presented a distinctly different pattern of depletion or very low enrichment for TFs across all cell types. Genes upregulated in both lineages, category UU, were further enriched for occupancy by TFs in both ERY and MEG beyond the more modest occupancy in HPC7. The induction of genes in the ERY and MEG lineages showed distinctive patterns. Genes induced specifically in ERY (categories NU and DU) were strongly enriched for occupancy by the examined TFs in erythroid cells but modest to no enrichment for TFs in HPC7 and MEG cells. This result indicates new binding by TFs at these genes during ERY differentiation. In contrast, genes induced specifically in MEG (categories UD and UN) were strongly enriched for most TF occupancy in *both* MEGs and HPCs.

The mapping of binding sites genome-wide for multiple TFs in HPC7 cells revealed coordinated binding by seven TFs—TAL1, LYL1, LMO2, ERG, FLI1, GATA2, and RUNX—and interactions among these proteins (47). Moreover, on analysis of mice that were compound heterozygotes for loss-of-function mutations, the *Gata2* and *Runx1* genes provided genetic evidence that the interplay between these TFs was needed for hematopoietic differentiation (47). The sites cobound by this TF heptad in HPC7 cells were enriched in most differential expression categories (Fig. 5*B*). Bar plots of the enrichment score for the TF heptad resolved the higher scores more clearly, and they showed a greater degree of enrichment of occupancy by the TF heptad for induction in MEG than in ERY (Fig. 5*C*). Using HPC7 data as a proxy for MEP, we infer from these results that many genes upregulated in MEG likely were already bound by TFs in MEP.

Genes downregulated in both lineages (category DD, Fig. 5*B*) and those repressed in the ERY lineage (categories ND and UD) were enriched for TF occupancy in the HPC line, but they were reduced for occupancy by many TFs in the erythroid and MEG cells. A similar trend was found for the DU category of genes repressed specifically in MEG, which was depleted for occupancy by GATA1 and had lower enrichment for other factors in MEG compared with HPC7. These patterns were consistent with activation of the genes by TF occupancy in HPC7, but the lineage-specific TFs in MEG did not replace those progenitor-bound factors, likely leading to repression during differentiation. The GATA and related factors provide striking examples of this TF loss. Binding by GATA2 in HPC7 cells was enriched in genes in the categories that were downregulated in at least one lineage (categories DD, ND, UD, and DU, not including DN), but binding by the paralogous GATA1 and the associated factor TAL1 was depleted upon repression in erythroid cells (DD, ND, and UD). Similarly, the occupancy for GATA1, TAL1, and FLI1 was less enriched or was depleted in MEG for repressed genes (categories DD and DU).

Taken together, these trends show that induction of genes in ERY frequently involved new binding of TFs, induction of genes in MEG occurred with retention or enhancement of TF binding compared with the pattern in HPC7 cells, and downregulation in both lineages was frequently accompanied by, and likely resulted from, loss of occupancy by key TFs.

### Discordance between chromatin- and RNA-based distances for MEP

**Figure 6:**
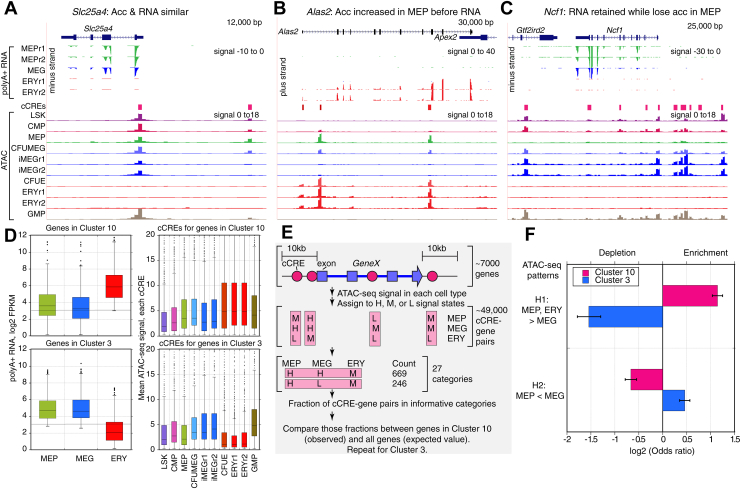
**Examples and patterns in genes with discordant chromatin accessibility and RNA patterns across MEP, MEG, and ERY.***A*–*C*, examples of genes showing either (*A*) concordance of RNA-Seq and ATAC-Seq patterns, (*B*) precocious actuation of cCREs inferred from ATAC-Seq compared with RNA-Seq patterns, or (*C*) high levels of RNA in MEP despite a low ATAC-Seq signal in cCREs. For each panel, the gene model from Gencode VM23 on the mm10 mouse genome assembly is shown at the *top*, followed by tracks of stranded RNA-Seq data, showing only the data on the transcribed strand (synonymous with the RNA). The *y*-axis for the signal is the same for all tracks in a panel; negative numbers are used for signal on the minus strand. The cCREs determined from normalized ATAC-Seq signals across mouse blood cell types from the VISION project (24) are shown above tracks of ATAC-Seq data for cell types in the differentiation series from the stem and progenitor population LSK, through CMP, MEP, precursors to MEG (CFUMEG), immature MEG (iMEG), precursors to ERY (CFUE), and ERY, and also including the progenitor for a different lineage, GMP, for comparison. The ATAC-Seq signal normalization was computed in 200 bp bins across the genome, and thus the signal (negative log base 10 of the *p* value for difference from expectation of a binomial distribution) was shown at a resolution of 200 bp. The same *y*-axis limits were set for signal across cell types. *D*, distributions of RNA-Seq signals for genes in differential expression clusters 10 and 3 and ATAC-Seq signals for cCREs associated with those genes. The distributions are summarized as box plots with the *box* extending from the first to the third quartile, the *line* indicating the median, and the *whiskers* extending to the most extreme data point that is no more than 1.5 times the interquartile range from the edge of the box, and with outliers shown as points. *E*, steps in compiling and categorizing cCRE–gene pairs for assessment of frequency of fit to hypothesized patterns to explain discordance in grouping of MEP with other cell types. *F*, assessment of the frequency of ATAC-Seq patterns predicted by two hypotheses (H1 and H2) to explain discordance in grouping of MEP with other cell types, comparing frequency of the patterns as observed for cCRE–gene pairs in clusters 10 and 3 with those for all genes as expected. The evaluation used Fisher’s exact test, with results expressed as an odds ratio, which was plotted as the log base 2 to distinguish enrichment (positive values) from depletion (negative values). The error bars cover the 95% confidence interval for the odds ratios. All comparisons were significant by Fisher’s exact test (*p* < 0.0000). Acc, chromatin accessibility; ATAC-Seq, assay for transposase-accessible chromatin followed by sequencing; cCRE, candidate *cis*-regulatory element; ERY, erythroblast; LSK, Lin-, Sca1+, Kit+; MEG, megakaryocyte; MEP, megakaryocyte–erythroid progenitor; r1 and r2, replicates 1 and 2.

The MEP population was grouped with other blood cell types differently depending on whether RNA or chromatin features were examined, with MEP clustering with multipotential progenitors and MEG by RNA distances but with ERY by chromatin accessibility measures (Fig. 1*A*). In many studies, chromatin accessibility and modifications such as H3K4 methylation have been strongly associated with RNA levels, and this pattern was observed for many genes in our work. For example, the gene *Slc25a4* is expressed in MEPs and MEGs but not erythroid cells, and ATAC-Seq peaks were observed at the transcription start site and upstream of the gene only in the cell types expressing this gene (Fig. 6*A*).

We reasoned that the discordance in cell type grouping could arise from sets of genes that showed a pattern of chromatin accessibility that differed from the RNA pattern across the three cell lineages. We searched for such examples in potentially informative clusters of genes defined by the robust differential expression assignments from the polyA+ RNA data, comparing the RNA levels with the normalized ATAC-Seq signals in blood cell types for cCREs from the VISION project (23, 24). One hypothesis to explain the greater similarity of MEP and ERY in chromatin accessibility compared with RNA was precocious actuation (accessibility to nucleases in chromatin) in MEP of cCREs for genes that would be expressed primarily in ERY. This hypothesis predicts that chromatin accessibility would be observed in the MEP population for genes with RNA levels that were low or undetectable in MEP but high in ERY. The genes in differential expression cluster 10 (Figs. 4, *C*, *D* and 6*D*) were candidates for manifesting the predicted pattern because they had low expression in MEP and MEG but high in ERY. Indeed, the *Alas2* gene fits with the predicted pattern of abundant RNA in ERY but not in MEP or MEG, coupled with unexpected strong ATAC-Seq peaks at cCREs in MEP, along with the expected ones in erythroid cells (Fig. 6*B*).

A second hypothesis to explain the discordance was that some genes expressed in MEP and MEG had low or undetectable ATAC-Seq signal at their associated cCREs in MEP, thereby leading MEP to be grouped with MEG by RNA data but not by chromatin accessibility. The genes in differential expression cluster 3 (Figs. 4 and 6*D*) were expressed in MEP and MEG but not in ERY, and therefore, they were candidates for manifesting the pattern predicted by the second hypothesis. The gene *Ncf1* conforms to the predicted pattern, with high RNA-Seq signals in MEP and MEG (not ERY) but low signal for chromatin accessibility in MEP along with high signal in MEG (Fig. 6*C*). Some of the genes with the discordance between levels of RNA and chromatin accessibility in MEP were in active chromatin in earlier multilineage progenitor cells, as illustrated by the ATAC-Seq signals in cCREs for *Ncf1* in LSK, CMP, and GMP (Fig. 6*C*). Possibly, these genes that were expressed in earlier progenitors, MEP and MEG, have lost chromatin accessibility in some of the subpopulations of MEP, perhaps those that are more likely to differentiate into erythroid cells, while retaining substantial stable polyA+ RNA.

We compared the patterns of RNA and chromatin accessibility more completely by collecting the sets of cCREs associated with each gene expressed in at least one of MEP, MEG, or ERY and tabulating the mean normalized ATAC-Seq signal in each of these three cell types using information in dbCRE from the VISION project (24). The cCREs found within the extended gene body were considered associated with a gene (Fig. 6*E*, see the Experimental procedures section). The distributions of ATAC-Seq signals in cCREs associated with genes in cluster 10 indicate some potential support for the hypothesis of precocious actuation of cCREs in MEP, with a trend toward higher ATAC-Seq signals in MEP than in MEG (Fig. 6*D*). The distribution of ATAC-Seq signals for cCREs associated with genes in cluster 3 indicates support for the second hypothesis, with a substantially lower distribution in MEP than in MEG (Fig. 6*D*). However, many different patterns of chromatin accessibility could be contributing to the observed broad distributions of signals.

We then adopted a categorization strategy to summarize an informative and comprehensive set of patterns for the ATAC-Seq signals in the three cell types. Three ATAC-Seq signal states, high (H), medium (M), and low (L), were defined using thresholds derived from the distributions of normalized ATAC-Seq signals in the mouse blood cell types (see the Experimental procedures section). Each of the 48,701 cCRE-gene pairs for the 7590 expressed genes was assigned to one of 27 possible categories of ATAC-Seq signal state across the three cell types (Table 1, Fig. 6*E*). This enumeration of cCRE–gene pairs in the signal states was used to test predictions of the two hypotheses to explain the discordance in cell type grouping by RNA *versus* chromatin accessibility. Each hypothesis predicted that cCRE–gene pairs would be in a particular subset of the chromatin state categories. The proportion of cCRE–gene pairs for all expressed genes that were assigned to that subset of categories served as the expected value, and the proportion of cCRE–gene pairs for genes in informative differential expression cluster 10 and in cluster 3 served as the observed values (Table 2; Fig. 6*E*). Specifically, we evaluated whether cCREs associated with genes in cluster 10 (high expression only in ERY) were enriched for cCREs precociously actuated in MEP (hypothesis 1) by calculating the proportion of cCRE–gene pairs in signal state categories with ATAC-Seq signals higher in MEP and ERY than in MEG. These cCREs for genes in cluster 10 were significantly enriched (odds ratio = 2.21), whereas the cCREs for genes in cluster 3 (high expression in MEP and MEG, not ERY) were significantly depleted (odds ratio = 0.34; Table 2; Fig. 6*F*). As a test of the second hypothesis, we evaluated whether cCREs associated with genes in cluster 3 were enriched for the predicted ATAC-Seq patterns with lower signal in MEP than in MEG. The cCREs associated with cluster 3 genes were significantly enriched for an ATAC-Seq signal higher in MEG than in MEP (odds ratio = 1.38), whereas the cCREs associated with cluster 10 genes were significantly depleted (odds ratio = 0.63; Table 2; Fig. 6*F*). All four comparisons were significant (*p* < 0.000) by Fisher’s exact test.

**Table 1:** Enumeration of cCRE–gene pairs in categories of ATAC-Seq patterns across cell types for enrichment analysis: Counts of all cCRE–gene pairs in categories

Cell type			
MEP	MEG	ERY	Number
H	H	H	3418
H	H	L	37
H	H	M	669
H	L	H	187
H	L	L	98
H	L	M	246
H	M	H	632
H	M	L	90
H	M	M	677
L	H	L	532
L	H	M	43
L	L	H	25
L	L	L	4549
L	L	M	730
L	M	H	3
L	M	L	8482
L	M	M	1051
M	H	H	1100
M	H	L	456
M	H	M	929
M	L	H	541
M	L	L	2825
M	L	M	3370
M	M	H	1366
M	M	L	4969
M	M	M	11,676
		Total	48,701

Tabulation of the 27 categories, which are combinations of high (H), medium (M), or low (L) ATAC-Seq signal at cCREs across cell types, plus counts for cCRE–gene pairs in those categories for all genes expressed in at least one cell type.

**Table 2:** Enumeration of cCRE–gene pairs in categories of ATAC-Seq patterns across cell types for enrichment analysis: Enrichment or depletion in informative categories

ATAC-Seq higher in MEP and ERY than MEG					
Cell type			Number of cCRE–gene pairs		
MEP	MEG	ERY	All genes	Cluster 10	Cluster 3
H	L	H	187	50	1
H	L	M	246	36	14
H	M	H	632	159	9
M	L	H	541	160	9
M	L	M	3370	645	118
Number pairs with higher signal in MEP and ERY			4976	1050	151
Total pairs in all genes or clusters			48,701	5223	4015
Percent			10.2	20.1	3.8
Odds ratio				2.21	0.34
95% Confidence interval				2.05–2.38	0.29–0.41
				Enriched	Depleted


ATAC-Seq low in MEP but medium or high in MEG					
MEP	MEG	ERY	All genes	Cluster 10	Cluster 3
L	H	H	0	0	0
L	H	L	532	19	71
L	H	M	43	7	2
L	M	H	3	0	0
L	M	L	8482	582	941
L	M	M	1051	132	50
Number pairs L in MEP, H or M in MEG			10,111	740	1064
Total pairs in all genes or clusters			48,701	5223	4015
Percent			20.8	14.2	26.5
Odds ratio				0.63	1.38
95% Confidence interval				0.58–0.68	1.28–1.48
				Depleted	Enriched

Enrichment or depletion of cCRE–gene pairs in categories predicted by hypothesized (B1) precocious actuation of cCREs in MEP or (B2) retention of RNA with loss of ATAC-Seq in MEP. The odds ratios and 95% confidence intervals were calculated using Fisher’s exact test.

Based on these analyses, we conclude that both (a) precocious actuation in MEP of cCREs for erythroid genes and (b) persistence of stable RNAs for some other genes in MEP despite a loss of chromatin accessibility contribute to the discordance in the grouping of MEP with other blood cell types depending on the experimental modality used to generate the distance metrics.

## Discussion

MEPs comprise a population of cells, derived from the CMP cell population, which is capable of differentiating into MEG and ERY. The MEPs do not represent a static, stable stage, but rather, they are a prominent intermediate population in the progressive differentiation from LSK through CMP and MEP to MEG and ERY. MEPs are not a homogeneous cell population, and human MEP can be resolved into at least three subpopulations. The RNA-Seq and ATAC-Seq data presented and examined in this article provided sensitive measurements, based on deep sequencing, of the composite transcriptomes and chromatin accessibility landscapes of the MEP population and of the more homogeneous ERY and MEG cell preparations. Examples of maintenance, repression, and induction of genes were found in both the lineages from MEP to either MEG or ERY. However, despite the confounding effects of heterogeneous subpopulations in MEP, we observed two distinct modes that dominate regulation in the two lineages.

Commitment to megakaryopoiesis features maintenance of an MEG program largely present in the MEP population, whereas erythroid differentiation requires a larger role of active induction and repression, that is, a rewiring of the regulatory program. The MEG and MEP transcriptomes were highly similar, as revealed by the shared expression of over twice as many genes between MEP and MEG than between MEP and ERY, the grouping of MEP and MEG to the exclusion of ERY in hierarchical clustering and SOMs, and the observation by PCA that the greatest variation in transcriptomes separated ERY from MEP and MEG. Analysis of differentially expressed genes revealed that differentiation to ERY involved the induction and repression of more genes than was found for differentiation to MEG. These conclusions about different modes of regulation in the two lineages were supported by analysis of TF occupancy, which showed that the genes differentially expressed on the lineage to MEG were already enriched for occupancy of key TFs in a cell line model for multipotent HPCs, whereas genes induced or repressed in the lineage to ERY showed large increases or decreases in enrichment, respectively, for induction and repression.

Our conclusions on the different modes of regulation strongly support earlier reports of maintenance of TF occupancy from HPCs to MEG in contrast to loss of occupancy at repressed genes and *de novo* occupancy at induced genes in ERY (19). Furthermore, analysis of transcriptomes and chromatin accessibility across diverse multipotent and maturing blood cell types in mice also showed substantial retention of transcripts and actuated cCREs from LSK through CMP to immature MEG, whereas significantly fewer progenitor-expressed transcripts and actuated cCREs were retained in ERY (22). The distinct programs of regulation in the MEG and ERY lineages were also supported by the observation that most genomic sites occupied by GATA1 and TAL1 differed between MEG and ERY (17, 19, 20). These different modes of regulation in the two lineages also include differences in DNA methylation, with substantial *de novo* DNA methylation during differentiation to MEG (22) in contrast to global demethylation of DNA during differentiation and maturation of ERY (22, 49). Despite the strong similarity in transcriptomes between MEP and MEG and the need for substantial regulatory rewiring in the ERY lineage, the high proliferative potential of the cells differentiating toward ERY leads to a substantial component of the MEP population committing to erythropoiesis, at least in humans (34).

Our data support some proposed processes in cellular differentiation. Several models of differentiation posit that cells with multilineage potential express many of the genes characteristic of the mature lineages, and specialization of cellular identity is achieved by progressive restriction of transcription (50, 51, 52). The broad transcription program observed in MEP and the repression of at least 1200 genes in the MEG and ERY lineages are consistent with this model. Among these MEP-expressed genes that were subsequently repressed were genes whose expression is characteristic of cells in other lineages, such as granulocytes and monocytes. Expression of those genes in MEP could reflect a cellular “memory” of greater potentiality at an earlier stage, such as CMP. Another process frequently observed is lineage priming (51), in which genes that are highly expressed in maturing lineages are also expressed at lower levels in the multipotent progenitor cells. For example, the MEP-expressed genes that continue expression in MEG but are repressed in ERY were highly enriched for MEG functions. Expression of many of these genes increased from MEP to MEG (such as cluster 4), thereby showing evidence for lineage priming. We also observed evidence of priming *via* chromatin accessibility, such as the genes in cluster 10 that were expressed only in ERY but which showed actuation of cCREs in MEP.

We conclude that MEPs have a strong bias, leaning toward an MEG fate at the expense of erythropoiesis. This MEG bias in MEP and reports that early hematopoietic stem and progenitor cells are biased toward an MEG fate fit with the molecular connections among platelets, hematopoietic stem cells, and endothelial cells, including similarity of cytokines or chemokine receptors expressed and the shared expression of TFs (53). However, differentiation of MEP to MEG is not a default program because the genome in differentiating MEG acquires substantial *de novo* methylation, which adds another layer of regulation during commitment and maturation to MEG (22).

Chromatin accessibility and histone modifications characteristic of active transcription or regulation, such as H3K4 methylation and H3K27 acetylation, are strongly associated with gene transcription. Indeed, these biochemical changes represent steps in the activation or memory of gene expression. However, multiple studies including ours have shown that the clustering of MEP with other hematopoietic cell types differs depending on the distance metric used, chromatin accessibility *versus* stable RNA accumulation. This unexpected discrepancy suggested that at least some sets of differentially expressed genes were showing an apparent dissociation between the acquisition of chromatin accessibility and activation of gene expression during differentiation of the MEP population to ERY or MEG. Our comparative analysis of chromatin accessibility and differential expression revealed two different categories of discordance between chromatin accessibility and gene expression. In the first category, regulatory elements were precociously actuated in MEP for genes that were silent in MEP but were highly expressed in ERY. The other category consisted of genes highly expressed in both MEP and MEG that showed little to no chromatin accessibility at their cCREs in MEP. These genes could include those that were expressed in earlier multipotent progenitor cells such as CMP, but they were no longer actively transcribed in MEP, reflected in a loss of chromatin accessibility, whereas their RNA was stable and hence still present in MEP. Genes in both categories would contribute to clustering of MEP with ERY by chromatin accessibility but clustering of MEP with MEG by RNA.

An issue for future study is to identify the molecular or cellular processes that lead to these discordances. One hypothesis is that such discordance is more likely to be observed during differentiation from progenitor cell populations, like MEP, with different subpopulations. For instance, the first category of apparently discordant genes might have cCREs highly accessible in a subpopulation of MEPs along with modest expression levels. In the heterogeneous MEP, this subpopulation could contribute measurably to the chromatin accessibility profiles but not to the RNA pool. This subpopulation may be biased toward commitment to the ERY lineage, which also would lead to substantial proliferation, which in turn would generate a high expression level of these genes in ERY. The second category of apparently discordant genes could reflect a high level of stable RNA in a subpopulation of MEP, perhaps from prior expression followed by loss of chromatin accessibility, or perhaps the genes continue expression in a low abundance subpopulation that did not contribute measurably to the chromatin accessibility profile. This general hypothesis of the role of subpopulations, illustrated by these scenarios, would predict that further refinement of robust subpopulations of MEP and increased sensitivity in multimodal single-cell analyses of these differentiation pathways would reveal stronger concordance in the chromatin accessibility and transcriptome profiles within subpopulations.

## Experimental procedures

### Cell isolation

Primary ERYs and MEGs were obtained from E14.5 CD-1 mouse fetal livers. ERY were isolated using immunoselection with TER119 antibodies, and MEGs were obtained by culturing KIT-positive cells for 12 days in a MEG expansion medium containing thrombopoietin followed by immunomagnetic sorting, as described previously (19). Cells from adult mouse bone marrow were fluorescence-activated cell sorted, selecting for Lin- (specifically Ter119-, CD11b-, Gr1-, IL7r1-, CD4-, CD8-, and B220-), KIT+, SCA1-, CD34^low^, CD16/32- cells, to obtain MEPs (37). Animal work followed the guidelines and policies of and was approved by the Animal Care and Use Committee of the National Human Genome Research Institute. The National Human Genome Research Institute is accredited by AAALAC International, and it operates in accordance with the Public Health Service Policy for the Care and Use of Laboratory Animals.

### Colony assays for MEPs

Mouse CFU-GM, CFU-mix, and BFU-E were assayed using Methocult, from Stem Cell Technologies (catalog no.: 3434). Mouse CFU-MKs were assayed using Megacult, from Stem Cell Technologies (catalog no.: 04974).

### RNA-Seq and read mapping

Total RNA was extracted from two independent biological replicates (5–10 million cells each) from each type using Qiagen RNeasy kits, followed by OligodT magnetic bead selection for polyA+ RNA. Strand-selective complementary DNA libraries were prepared using dUTP in place of dTTP in second-strand complementary DNA synthesis to allow its subsequent, selective digestion (39). Libraries were sequenced on the Illumina HiSeq 2000 to a minimum depth of 100 million read pairs per sample. Reads were mapped to the mouse mm9 reference genome using the spliced read aligner TopHat2 (54, 55), which was supplied with Illumina's iGenomes mm9 RefSeq GTF as a gene model reference. A two-step mapping strategy (56) was used to obtain and combine splice junctions from all samples that could be used to annotate mapped reads. The first round of mapping identified all novel splice junctions in each replicate, which were then combined across samples to obtain a master set of combined novel junctions. A second round of mapping was performed, with the master set of junctions supplied using option "-j" (--raw-juncs). At this step, "--no-novel-juncs" was enabled so that all mapped reads were annotated with splice junctions only from this master set, thereby obtaining assembly based on an all-inclusive set of splice junctions derived from all the samples. The sorted BAM output from the second round of mapping was used as input to Cufflinks (40, 41, 42) for quantification of expression levels for individual replicates and to Cuffdiff (43) for differential expression testing. The coverage across exons was calculated, and signal tracks were generated from this BAM output using SAMTools (57), BEDTools (58), the UCSC Table Browser (59), and other UCSC utilities (60, 61, 62, 63).

### Quantification of expression levels

We used a custom gene model annotation file in which each gene was represented by a single canonical transcript to estimate expression levels for RefSeq genes. Starting with an Illumina iGenomes RefSeq mm9 GTF, we obtained canonical transcripts for each gene from the "knownCanonical" table using the UCSC Table Browser (59) to represent that gene. For genes without any record of a canonical transcript, we chose a representative transcript based on transcript length (longest), coding sequence length (longest), and number of exons (greater). We excluded genes positioned on chrN_random or chrUn_random and snoRNAs matching the pattern "Snora." This resulted in 22,977 genes, each with a single transcript. We used Cuffdiff (40, 41, 42) to identify differentially expressed genes, using this custom GTF with the parameters --dispersion-method set to per-condition, --library-type set to fr-firststrand, --max-bundle-frags = 20,000,000, --min-reps-for-js-test = 2, -b for bias correction, and –M to mask globin transcripts. However, regions on mouse chr11 and chr7 containing alpha-globin and beta-globin gene transcripts were masked from Cuffdiff, using option -M (see section on "*Globin expression estimation*" below). Transcript abundance levels pooled across replicates were expressed in terms of log2-transformed FPKMs, after addition of a value of 1.1 as noise. Noise addition was done to avoid log-transforms of zero values and divide-by-zero issues. Thus, genes with FPKM of 0 are log2-transformed to an expression level of 0.1375. Genes whose transcripts passed the FDR 0.05 threshold and were above our threshold for expression (log2 FPKM >3) in both cell types being compared were considered to be differentially expressed.

### Globin gene expression estimation

Globin genes are expressed in enormous amounts in erythroid cells. Despite the availability of high-performance compute clusters, estimating abundances for globin mRNAs and performing differential expression tests at these loci is time and memory intensive, and programs often do not complete running, depending upon the number of reads. To avoid these issues, we (bioinformatically) masked the alpha and beta globin loci on chr11 and chr7, while estimating expression levels and performing differential expression tests for the other genes using Cuffdiff. This is done by supplying a GTF with globin loci to Cuffdiff, using the option -M/--maskfile. As a result, all alpha and beta globin genes, including fetal globins, were reported as not differentially expressed, with an FPKM of 0. To obtain a measure of expression for globins, we extrapolated the expected FPKM of globins from their read counts, by comparing read counts and FPKMs of the top 20 highly expressed genes. The ratio of read counts to FPKMs (∼2 in this case) was used to estimate globin gene expression levels. Therefore, the reported expression level of globin genes is not from Cuffdiff.

### Pseudoreplicate approach for MEG

To avoid the low number of alignments for MEG replicate 2 affecting our analyses and differential expression tests, we used pseudoreplicates for the MEG sample. Pseudoreplicates were generated by pooling alignments from both MEG replicates and randomly splitting the pooled alignments into datasets with equal numbers of alignments. Pseudoreplicate expression levels from Cufflinks (each replicate separately) and Cuffdiff (pooled values from pseudoreplicates) were used for analysis and to perform pairwise differential expression tests. This approach underestimates the real variance in the data, so to avoid potential false discoveries during differential expression testing; we used a more stringent FDR of 0.02 for the identification of differentially expressed genes specifically for the MEP *versus* MEG comparison.

### Functional term enrichments

Functional term enrichments were computed using the GREAT tool (46) by using 20 bp upstream of the promoter of each gene as input. Genes from each of the differential expression categories were examined separately, and terms with enrichments at or below a binomial FDR <0.05 were retained. This produced a list of 1634 terms from multiple ontologies enriched in at least one of the differential expression gene clusters (Table S2). Redundancies in this list were removed to generate a set of 200 terms covering the common themes in the enriched terms. A subset of these terms was extracted to emphasize the five major classes of terms presented in Figure 5*A* (Table S2).

### Enrichment of occupancy by TFs

For each group of genes, we calculated the ratio of % occupancy by a TF in that expression category to the % occupancy by the same TF in the genomic background (all 1239 nondifferentially expressed genes). These values were then log2-transformed to represent enrichment (positive values) and depletion (negative values).

### Categorization of cCREs by ATAC-Seq signal state across cell types

A set of cCREs was considered associated with a gene, that is, potentially involved in its regulation, if the elements in that set occurred within a genomic interval extending from 10 kb upstream of the transcription start site to 10 kb downstream of the polyA addition site of the gene. For all 7590 genes expressed in at least one of the three cell types, we collected the associated cCREs to generate 48,701 cCRE–gene pairs. In situations of close or overlapping genes, a cCRE could be assigned to more than one gene. We tabulated the mean normalized ATAC-Seq signal in mouse blood cell types using information in dbCRE from the VISION project (24). Specifically, we used the negative logarithm (base 10) of the *p* value for deviation from a negative binomial distribution as the quantitation for the normalized ATAC-Seq signal. The distribution of ATAC-Seq signals was evaluated in a subset of the mouse blood cell types, which included the hematopoietic stem and multilineage progenitor cells LSK, CMP, MEP, and GMP, the lineage-restricted progenitor cells CFUMEG and CFUE, and lineage-restricted immature cells, MEG and ERYs. The distribution of these normalized ATAC-Seq signals was compared with the distribution of RNA-Seq signals (log2 FPKM) for the polyA+ RNA-Seq for MEP, MEG, and ERY in Figure 6*D*. For all comparisons of patterns of signals between transcriptomes and chromatin accessibility, we used the transcriptome data from polyA+ RNA-Seq in mature MEG and chromatin accessibility from ATAC-Seq data in immature MEG because no ATAC-Seq data were available for mature MEG. The cCRE–gene pairs along with gene expression levels and normalized ATAC-Seq signals at the cCREs in each cell type are provided in Table S3.

To systematically examine the patterns of chromatin accessibility across cell types, we assigned the ATAC-Seq signals in MEP, immature MEG, and ERY to signal states using thresholds derived from the distributions of ATAC-Seq signal strengths. We previously set a value of 1.3 (negative logarithm [base 10] of the *p* value) as the threshold for calling an ATAC-Seq peak (24). Any cCRE with an ATAC-Seq signal value below 1.3 in a cell type was assigned to the low (L) signal state in that cell type. The mean of the ATAC-Seq signal across all genes and cell types in the current study was 8.6, and this value was chosen to assign cCREs to the midrange (M, below 8.6) *versus* high (H, at least 8.6) signal states in each cell type. The 27 possible combinations of H, M, or L signal states in three cell types gave 27 categories of signal state across cell types (Table 1). Each of the 48,701 cCRE–gene pairs was assigned to an ATAC-Seq signal state.

### Tests of predictions from hypotheses to explain discordance in cell type grouping

Predictions of the hypotheses for explaining discordance in cell grouping between RNA and chromatin accessibility measures were tested by evaluating the observed *versus* expected frequencies of cCRE–gene pairs in informative groups of categories of ATAC-Seq signal states (Table 2). The observed values were the numbers of cCRE–gene pairs in informative signal state categories for the genes in differential expression clusters 10 or 3, and the expected values were the numbers of cCRE–gene pairs in informative signal state categories for the 7590 genes expressed in any of the three cell types. The data were arranged in 2 × 2 contingency tables to compare the numbers of cCRE–gene pairs in or not in the informative signal states for genes in a differential expression cluster (observed) *versus* the numbers of cCRE–gene pairs in or not in the informative signal states for all expressed genes (expected). The odds ratio, 95% confidence interval, and *p* values for deviations from expectation were computed using Fisher’s exact test. For testing a prediction from the hypothesized precocious actuation of cCREs in MEP for some ERY-expressed genes, the informative ATAC-Seq signal state categories had a higher signal in MEP and ERY than in MEG (Table 2). For testing a prediction from the hypothesized loss of accessibility at cCREs in MEP for some MEG-expressed genes, the informative ATAC-Seq signal state categories had a low signal in MEP, medium or high in MEG, and any value in ERY (Table 2).

## Data availability

Data are available in the Gene Expression Omnibus as accessions GSE40522 for RNA-Seq, GSE51338 for ChIP-Seq data, and GSE143271 and GSE229101 for chromatin accessibility data. These data are also available at the ENCODE Project data portal (https://www.encodeproject.org). Data can be visualized using track hubs from the VISION project website (https://usevision.org) or on a customized browser (http://main.genome-browser.bx.psu.edu).

## Supporting information

This article contains supporting information (19, 22, 23, 24, 30, 31, 47, 48).

## Conflict of interest

M. J. W. is a consultant for GlaxoSmithKline, Cellarity, Graphite Bio, Fulcrum Therapeutics, and Dyne Therapeutics and an equity owner in Cellarity. The other authors declare that they have no conflicts of interest with the contents of this article.
