# Supplementary material for: Transcriptome and chromatin accessibility divergence during differentiation of a bipotential progenitor cell population to erythroblasts and megakaryocytes
Source: J Biol Chem. 2025 Oct 14;301(12):110819. doi: 10.1016/j.jbc.2025.110819 (PMC12664380; doi:10.1016/j.jbc.2025.110819)
Supplement: Supporting Figures [file mmc1.pdf]

Supporting Information for:

Transcriptome and chromatin accessibility divergence during differentiation of a bipotential progenitor cell population to erythroblasts and megakaryocytes

Tejaswini Mishra, Belinda M. Giardine, Christapher S. Morrissey, Cheryl A. Keller, Elisabeth F. Heuston, Stacie M. Anderson, Vikram R. Paralkar, Maxim Pimkin, Mitchell J. Weiss, David M. Bodine and Ross C. Hardison

Table of Contents

Item	Description	Page
Table of Contents		0
Supporting Figure S1	Grouping of MEP with different cell types	1
Supporting Figure S2	RNA-seq data and processing	2
Supporting Figure S3	Choice of *k* number of clusters	3
Supporting Figure S4	Peaks and sources of TF occupancy data	4
Supporting Table 1 Legend	Gene expression levels and differential expression assignments	5
Supporting Table 2 Legend	Function related terms for differential expression clusters	6
Supporting Table 3 Legend	cCRE-gene pairs with gene expression and ATAC-seq signal	7-8

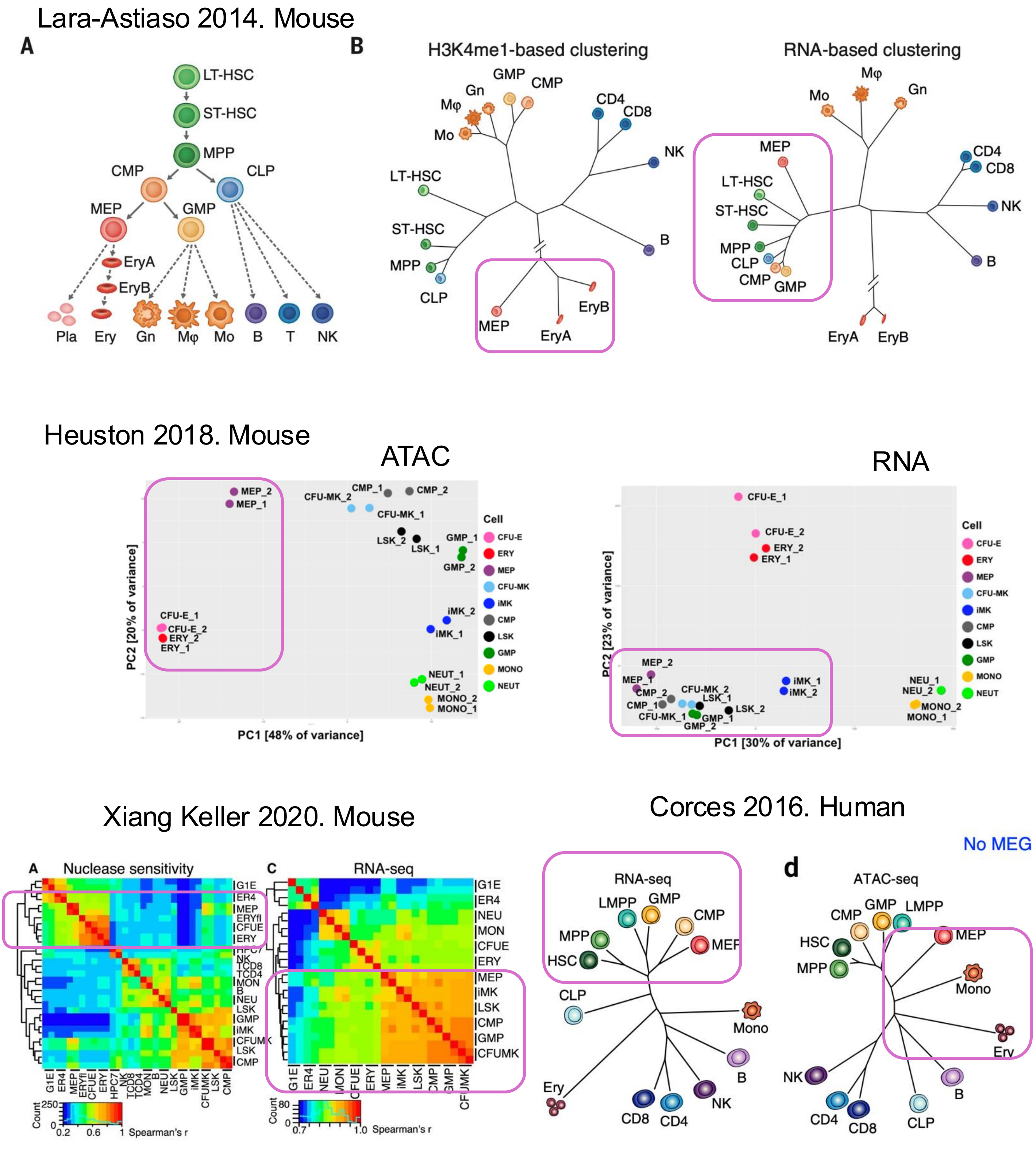

Supplemental Figure S1. Published results showing MEP grouping with MEG and multilineage progenitor cells when using RNA levels as a distance metric but MEP grouping with ERY when using chromatin accessibility or histone modification (H3K4me1) as the distance metric. These images were taken from the published figures and annotated with rounded rectangles to emphasize the discordance. These results were synthesized in the summary shown in Figure 1A. The papers are Lara-Astiaso 2014 (reference 30), Heuston 2018 (reference 22), Xiang Keller 2020 (reference 23), and Corces 2016 (reference 31).

A

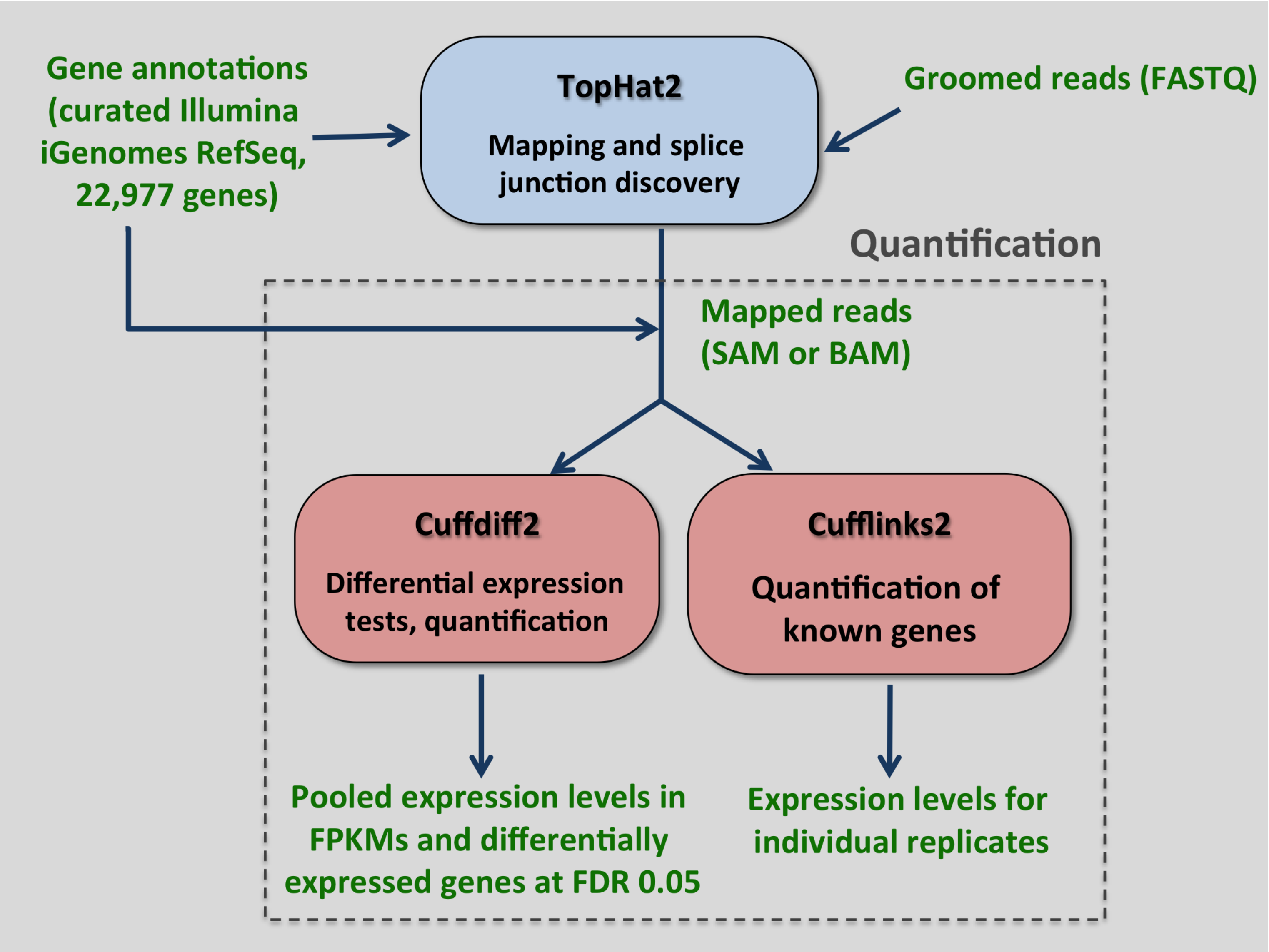

B

	# Read Pairs	# Mapped Reads	# Alignments	% Mapped Reads
MEP Rep1	2 x 123,922,076	94,308,729	180,399,326	76.1%
MEP Rep2	2 x 120,129,876	84,178,334	161,120,323	70.1%
ERY Rep1	2 x 117,566,409	61,078,428	115,103,757	52%
ERY Rep2	2 x 106,890,312	49,524,755	89,069,700	46.3%
MEG Rep1	2 x 107,938,301	58,026,025	112,235,879	53.8%
MEG Rep2	2 x 101,667,130	3,491,255	7,332,882	3.4%

Supplemental Figure S2. RNA-seq data processing (A) Schematic of the RNA-seq analysis pipeline. Tools used are in colored, solid boxes. Input and output files and data are indicated in green. (B) RNA-seq read mapping statistics. For each sample, the number of sequenced reads, mapped reads, and alignments, as well as percentage of mapped reads are shown.
Col. 1: # Read Pairs = number of paired sequence reads, where each read in a pair has the same read ID
Col. 2: # Mapped Reads = number of unique read IDs with at least one end mapped
Col. 3: # Alignments = number of genomic locations ("hits to the genome") the reads mapped to, when allowing multiple mapping.
Col. 4: % Mapped Reads = (Col. 2/Col.1)*100

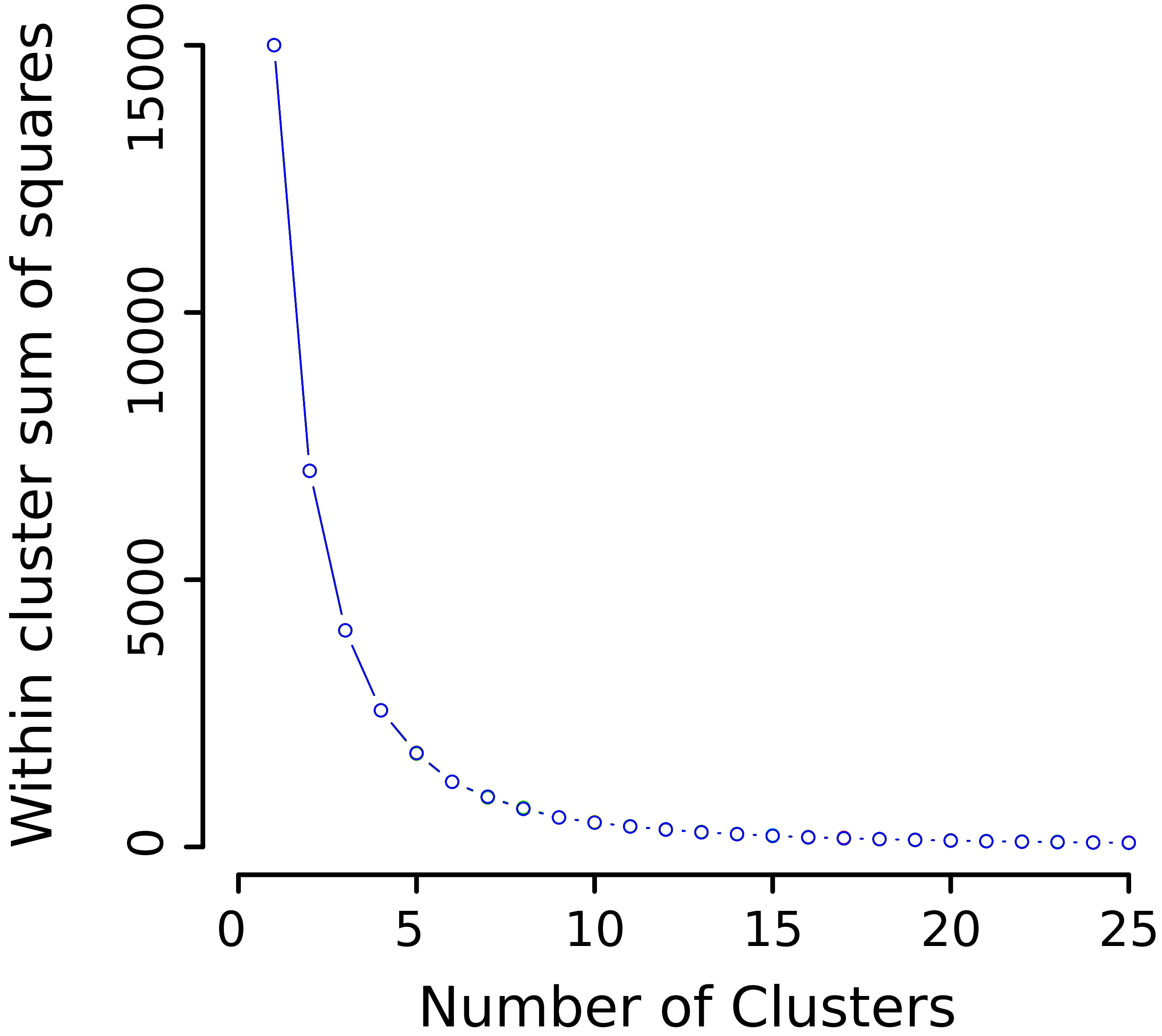

Supplemental Figure S3. Choice of "k" or number of clusters. K-means clustering was performed on standardized gene e pression levels for k ranging from 1 to 25, and the within cluster sum-of-squares (WCSS) was calculated for each value of "k". This procedure was performed 20 times. The plot shows the WCSS (hollow circles) obtained at each value of "k", for each of the 20 iterations. The "k" at which the within cluster sum-of-squares did not decrease drastically with an increase in number of clusters (k = 12) was chosen as the optimal number of groups to partition genes into.

TF	# Peaks	Cell type	Source
GATA1	5767	TER119+ fetal liver erythroblasts	Pimkin et. al.
TAL1	3086	TER119+ fetal liver erythroblasts	Pimkin et. al.
ETO2	1455	DMSO Induced MEL	Soler et. al.
MTGR1	4682	DMSO Induced MEL	Soler et. al.
GATA1	1727	CD41+ fetal liver megakaryocytes	Pimkin et. al.
GATA2	2728	CD41+ fetal liver megakaryocytes	Pimkin et. al.
TAL1	3505	CD41+ fetal liver megakaryocytes	Pimkin et. al.
FLI1	2001	CD41+ fetal liver megakaryocytes	Pimkin et. al.
GATA2	9099	HPC-7	Wilson et. al.
LMO2	9006	HPC-7	Wilson et. al.
LYL1	4196	HPC-7	Wilson et. al.
RUNX1	4988	HPC-7	Wilson et. al.
TF Heptad	1007	HPC-7	Wilson et. al.

Supplemental Figure S4: Peak statistics and sources of TF occupancy data

Legend for Supporting Information, Table 1
Gene expression levels and differential expression assignments

For each gene (row), the columns give information on chromosome coordinates, level of expression, and assignments of differential expression. Specifically, the columns are:

gene Name of the gene
chrom Name of the chromosome
TSS Position of the transcription start site
chromStart Beginning of the gene annotation, mm9 coordinates
chromEnd Ending of the gene annotation, mm9 coordinates
MEP Expression level in MEP as measured by polyA+ RNA-seq, units are log2(FPKM)
MEG Expression level in MEG as measured by polyA+ RNA-seq, units are log2(FPKM)
ERY Expression level in ERY as measured by polyA+ RNA-seq, units are log2(FPKM)
MEPvsMEG Differential expression between MEP and MEG
MEPvsERY Differential expression between MEP and ERY
MEGvsERY Differential expression between MEG and ERY
For the previous 3 columns, cell entries mean:
1 = upregulated in CellType2 vs. CellType1
-1 = downregulated in CellType2 vs. CellType1
0 = not significantly changing
ExpressedAny Expressed in any of the three cell types
ExpressedMEP Expressed in MEP
ExpressedMEG Expressed in MEG
ExpressedERY Expressed in ERY
For the previous 4 columns, cell entries mean:
1 = expressed
0 = not expressed
kmeansClusNum The *k*-means cluster number to which a differentially expressed gene is assigned; Figure 4C
ExpCat.CuffDiff The expression category to which a gene is assigned by CuffDiff
ExpCat.kmeans The expression category to which a gene is assigned by *k*-means
Consensus The expression category to which a gene is assigned using a consensus between CuffDiff and *k*-means assignments
For the previous 3 columns, cell entries are:
UU, UN, UD, NU, DU, NN, DN, ND, DD: U is up-regulated, D is down-regulated, and N is no significant change measured. The first letter refers to the differentiation from MEP to MEG, the second letter refers to the differentiation from MEP to ERY
XX = not in consensus set, i.e. when *k*-means and Cuffdiff calls did not match

Legend for Supporting Information, Table 2
Function related terms for genes in differential expression clusters

Functional term enrichments were computed using the Genomic Regions Enrichment of Annotations Tool (GREAT). Groups of genes from each of the differential expression categories were examined separately, and terms with enrichments at or below a binomial FDR < 0.05 were retained. This produced a list of 1634 terms from multiple ontologies enriched in at least one of the differential expression gene clusters, listed in the tab “AllSignificantResultsFromGREAT” in the Excel workbook Supporting Information Table 2. Redundancies in this list were removed to generate a set of 200 terms covering the common themes in the enriched terms, listed in the tab “200Terms_LogQ”. A subset of these terms was extracted to emphasize the five major classes of terms presented in Figure 5A; these are in the tab “OrganizedForFigure”.

For the tabs “AllSignificantResultsFromGREAT” and “200Terms_LogQ”, each row starts with an Ontology category and a function-related term within that category (column A), in the format “Ontology_*_Term”. The next 9 columns (B-J) list the FDR Q-value (binomial test) for the enrichment of the function-related term within the specified group of genes. Each of the columns B-J gives those Q-values for a set of genes in a category of differential expression. Specifically, the categories are UU, UN, UD, NU, DU, NN, DN, ND, DD, where U is up-regulated, D is down-regulated, and N is no significant change measured. The first letter refers to the differentiation from MEP to MEG, the second letter refers to the differentiation from MEP to ERY. The column headings are colored red for categories of genes up-regulated during differentiation from MEP to ERY (NU and DU), light blue for categories of genes that are up-regulated during differentiation from MEP to MEG (UN and UD), green for categories of genes that are down-regulated or not changing upon differentiation of MEP to either MEG or ERY (DD, ND, and DN), a darker blue for the category that is up-regulated along both branches (UU) and yellow for the category that is down-regulated along both branches (DD). NA means not applicable, i.e., the Ontology_*_Term was not enriched in that set of genes.

In the tab “200Terms_LogQ”, the enrichments are expressed as the negative log (base 10) of the Q-values. These values are shaded green using conditional formatting.

In the tab “OrganizedForFigure”, an additional descriptor for “Class of terms” was added to describe the specific cell type or process illustrated by the selected terms.

Legend for Supporting Information, Table 3
cCRE-gene pairs with gene expression and ATAC-seq signal

Each row describes a cCRE-gene pair, *i.e.* an annotated gene along with an associated candidate *cis*-regulatory element (cCRE). A cCRE was considered to be associated with a gene if it was located within a genomic interval extending from 10kb upstream of the transcription start site to 10kb downstream of the polyA addition site of the gene. Multiple cCREs could be associated with a single gene, and thus the same gene can be included on multiple rows with a different associated cCRE on each row.

Additional columns on each row provide information about the expression of the gene and the level of chromatin accessibility, inferred from ATAC-seq data, of the cCRE across a set of mouse blood cell types, including multi-lineage progenitor cells, differentiated cells and mature cells. The expression data repeats some information from Supporting Information Table 1, but it also has genome coordinates on the mm10 assembly, which were needed to combine with the ATAC-seq signals, which were also on mm10. The ATAC-seq signal levels were obtained from the normalized data in the VISION project (Xiang et al. 2024. Genome Research 34:1089-1105).

Specifically, the columns are:
geneChrMm10 Chromosome for gene, mm10 assembly
st start position of annotation for the gene on mm10
end ending of annotation for gene on mm10
name Gene name
chromMm9 Chromosome for gene, mm9 assembly
mm9tss Position of the transcription start site, mm9 assembly
mm9st Beginning of the gene annotation, mm9 coordinates
mm9end Ending of the gene annotation, mm9 coordinates
MEP Expression level for gene in MEP as measured by polyA+ RNA-seq, units are log2(FPKM)
MEG Expression level in MEG as measured by polyA+ RNA-seq, units are log2(FPKM)
ERY Expression level in ERY as measured by polyA+ RNA-seq, units are log2(FPKM)
MEPvsMEG Differential expression between MEP and MEG
MEPvsERY Differential expression between MEP and ERY
MEGvsERY Differential expression between MEG and ERY
For the previous 3 columns, cell entries mean:
1 = upregulated in CellType2 vs. CellType1
-1 = downregulated in CellType2 vs. CellType1
0 = not significantly changing
ExpressedAny Expressed in any of the three cell types
ExpressedMEP Expressed in MEP
ExpressedMEG Expressed in MEG
ExpressedERY Expressed in ERY
For the previous 4 columns, cell entries mean:
1 = expressed
0 = not expressed
kmeansClusNum The *k*-means cluster number to which a differentially expressed gene is assigned; Figure 4C
ExpCat.CuffDiff The expression category to which a gene is assigned by CuffDiff
ExpCat.kmeans The expression category to which a gene is assigned by *k*-means

Consensus The expression category to which a gene is assigned using a consensus between CuffDiff and *k*-means assignments

For the previous 3 columns, cell entries are:

UU, UN, UD, NU, DU, NN, DN, ND, DD: U is up-regulated, D is down-regulated, and N is no significant change measured. The first letter refers to the differentiation from MEP to MEG, the second letter refers to the differentiation from MEP to ERY

XX = not in consensus set, i.e. when *k*-means and Cuffdiff calls did not match

The next four columns give chromosomal coordinates for the cCRE in each cCRE-gene pair.

ccreChr Chromosome for cCRE, mm10 assembly
ccreSt start position of annotation for the cCRE on mm10
ccreEnd ending of annotation for cCRE on mm10
ccrename Name of the cCRE, in the format chromosome_startPosition_endPosition

The remaining columns give the normalized ATAC-seq signal for the designated cCRE in multiple mouse blood cell types. These values are for signals in:

AVE Average ATAC-seq signal across cell types
B_r1 ATAC-seq signal in B -ells
CFUE_r1 ATAC-seq signal in colony forming units erythroid
CFUMK_r1 ATAC-seq signal in colony forming units megakaryocyte
CMP_r1 ATAC-seq signal in the common myeloid progenitor cell population
ER4_r1 ATAC-seq signal in the cell line G1E-ER4, with activated GATA1-ER, a model for an early stage of maturing erythroblasts
ER4_r2 ATAC-seq signal in the cell line G1E-ER4, with activated GATA1-ER, a model for an early stage of maturing erythroblasts
ERY_ad_r1 ATAC-seq signal in erythroblasts from adult bone marrow
ERY_ad_r2 ATAC-seq signal in erythroblasts from adult bone marrow
ERY_fl_r1 ATAC-seq signal in erythroblasts from fetal live
G1E_r1 ATAC-seq signal in the cell line G1E, an erythroid line with no GATA1, and a model for an early erythroid progenitor cell
G1E_r2 ATAC-seq signal in the cell line G1E, an erythroid line with no GATA1, and a model for an early erythroid progenitor cell
GMP_r1 ATAC-seq signal in the granulocyte monocyte progenitor cell population
HPC7_r1 ATAC-seq signal in the cell line HPC7, a model for a multi-lineage hematopoietic progenitor cell
LSK_r1 ATAC-seq signal in the hematopoietic stem cell population
MEL_r1 ATAC-seq signal in the murine erythroleukemia cell line, a model for immature erythroblasts
MEL_r2 ATAC-seq signal in the murine erythroleukemia cell line, a model for immature erythroblasts
MEP_r1 ATAC-seq signal in the megakaryocyte-erythroblast progenitor cell population
MON_r1 ATAC-seq signal in monocyte
NEU_r1 ATAC-seq signal in neutrophils
NK_r1 ATAC-seq signal in natural killer cells
T_CD4_r1 ATAC-seq signal in CD4+ T-cells
T_CD8_r1 ATAC-seq signal in CD8+ T-cells
iMK_r1 ATAC-seq signal in immature megakaryocytes
iMK_r2 ATAC-seq signal in immature megakaryocytes

r1 = replicate 1, r2 = replicate 2
